# Too Much SAMA, Too Many Exacerbations: A Call for Caution in Asthma

**DOI:** 10.3390/jcm14145046

**Published:** 2025-07-16

**Authors:** Fernando M. Navarro Ros, José David Maya Viejo

**Affiliations:** 1 Centro de Salud Plaza Segovia, Plaza Segovia s/n, 46017 Valencia, Spain; fnavarroyros@hotmail.com; 2 Centro de Salud de Camas, Santa Maria de Gracia 54, Camas, 41900 Seville, Spain

**Keywords:** asthma, short-acting muscarinic antagonist (SAMA), short-acting β_2_-agonist (SABA), asthma exacerbations, systemic corticosteroids, healthcare utilization, treatment guidelines, reliever overuse

## Abstract

**Background/Objectives:** The overuse of short-acting β_2_-agonists (SABAs) has been associated with increased asthma morbidity and mortality, prompting changes in treatment guidelines. However, the role of frequent short-acting muscarinic antagonists (SAMAs) use remains poorly defined and unaddressed in current recommendations. This study offers the first real-world analysis of SAMA overuse in asthma, quantifying its association with exacerbation risk and healthcare utilization and comparing its predictive value to that of SABAs. **Methods:** A retrospective multicenter cohort study analyzed electronic health records (EHRs) from 132 adults with asthma in the Spanish National Health System (SNS). Associations between annual SAMA use and clinical outcomes were assessed using negative binomial regression and 5000-sample bootstrap simulations. Interaction and threshold models were applied to explore how SAMA use affected outcomes and identify clinically actionable cutoffs. **Results:** SAMA use was independently associated with a 19.2% increase in exacerbation frequency per canister and a nearly sixfold increase in the odds of experiencing ≥1 exacerbation (OR = 5.97; 95% CI: 2.43–14.66). An inflection point at 2.5 canisters/year marked the threshold beyond which annual exacerbations exceeded one. Increased SAMA use was also associated with a higher number of respiratory consultations and with more frequent prescriptions of systemic corticosteroids and antibiotics. The risk increased more sharply with SAMAs than with SABAs, and the lack of correlation between them suggests distinct clinical patterns underlying their use. **Conclusions:** SAMA use emerges as a digitally traceable and clinically meaningful indicator of asthma instability. While the associations observed are robust and consistent across multiple outcomes, they should be considered provisional due to the study’s retrospective design and limited sample size. Replication in larger and more diverse cohorts is needed to confirm external validity. These findings support the integration of SAMA tracking into asthma management tools—alongside SABAs—to enable the earlier identification of uncontrolled disease and guide therapeutic adjustment.

## 1. Introduction

Asthma is one of the most prevalent and burdensome chronic diseases worldwide, affecting approximately 262 million individuals and consuming substantial healthcare resources [1,2,3,4]. Although substantial therapeutic advances have been achieved in recent decades, poor asthma control remains common—often under-recognized—and is frequently driven by sustained reliever overuse. As airway inflammation is the central pathological process in asthma, the use of treatments that do not target this mechanism undermines effective disease management [5,6].

Reliever medications are designed to provide rapid symptom relief and are intended for use on an as-needed basis to reverse or prevent bronchoconstriction [7,8,9]. These include short-acting β_2_-agonists (SABAs) and short-acting muscarinic antagonists (SAMAs). While SABAs have been extensively studied as monotherapy, recent findings from large observational programs—such as the SABINA studies—have linked their frequent use to poor clinical outcomes, including higher exacerbation rates and increased mortality risk [7,8,10,11,12,13,14,15,16,17,18,19]. As a result, major international guidelines (GINA 2025, GEMA 5.4, NICE 2024) currently discourage the use of SABA monotherapy and instead recommend appropriate maintenance therapy with inhaled corticosteroids, combined with rescue treatment using either SABAs or anti-inflammatory relievers [7,8,10,17,19,20].

SAMAs, however, have received comparatively little attention despite their routine use in clinical practice, often as an alternative for patients who experience adverse effects from SABAs or have a poor response to β_2_-agonists [21,22].

Their omission from asthma treatment algorithms does not imply established safety but rather reflects a lack of robust evidence [7,8,17]. Pharmacologically, SAMAs share key characteristics with SABAs, including a short duration of action (approximately 4–6 h), rapid onset (though slower than SABAs), and no anti-inflammatory effect [7,8,22]. Nevertheless, the long-term clinical implications of SAMA overuse remain poorly understood.

Emerging evidence suggests that the number of reliever inhalers prescribed—both SABAs and SAMAs—may reflect poor asthma control [23]. While the association between SABA overuse and worse outcomes is well established, the prognostic value of SAMA use remains largely unexplored. This knowledge gap limits the ability of clinicians to interpret SAMA prescribing patterns and to identify patients at risk of deterioration.

This study aims to address this issue by examining the clinical relevance of SAMA overuse in asthma. We hypothesize that excessive SAMA use may mirror the risk profile of SABA overuse and potentially serve as an earlier or more sensitive marker of poor disease control in specific patient subgroups. To test this hypothesis, we analyzed real-world data from a large primary care cohort of patients with asthma, with the following objectives:

1. To quantify the association between SAMA use and asthma exacerbation frequency.
2. To identify a clinically relevant threshold of SAMA overuse.
3. To compare dose–response risk patterns for SAMAs versus SABAs.
4. To assess whether SAMA use predicts outcomes independently of other treatment factors.

The findings may help to identify earlier uncontrolled asthma and support the development of smarter prescribing by clinical alerts and decision-support tools [24].

## 2. Materials and Methods

### 2.1. Study Design and Data Source

This was a retrospective and multicenter cohort study based on anonymized electronic health records (EHRs) from two primary care centers in Spain (Valencia and Seville), both integrated into the Spanish National Health System (SNS). The study is part of the Seleida project, which identifies the role of SAMA use as a predictive marker of asthma instability in real-world settings [23]. This analysis was conducted as a pilot study, with the sample size determined pragmatically based on feasibility and data availability at the participating centers.

The study protocol was approved by the relevant ethics committees and registered on national biomedical research ethics platforms [23].

### 2.2. Data Source and Patient Selection

The sample included 132 adults with asthma, selected via stratified random sampling from EHRs extracted between 1 May and 31 July 2024. For each patient, clinical and pharmacological data from the 12 months prior to inclusion were collected to support a comprehensive evaluation of medication exposure and outcomes. A random number table was used to select individuals from a pre-filtered list of 5874 patients—drawn from a general population of 82,631—who met the inclusion criteria after applying predefined exclusion criteria.

Each patient was assigned a unique, anonymized identifier in compliance with European data protection regulations. Informed consent was waived due to the retrospective, non-interventional nature of the study and the use of anonymized data. No patient was contacted, and no clinical decision was influenced by the study. All data handling procedures complied with national data protection regulations.

#### 2.2.1. Inclusion Criteria

Eligible patients were adults aged 18 to 80 years with a confirmed diagnosis of asthma based on clinical evaluation and/or spirometric data. In recognition of diagnostic variability in real-world settings, patients without a formal EHR-coded diagnosis were also included if they had received asthma-specific pharmacological treatment for a minimum of three months per year over the past two years.

#### 2.2.2. Exclusion Criteria

To reduce clinical heterogeneity and potential confounding, patients classified as step 6 according to GEMA 5.4 were excluded, given the advanced therapeutic complexity associated with this category. Additional exclusions comprised individuals with active malignancies, those receiving palliative care, pregnant women, and patients receiving biologic agents or long-term systemic corticosteroids for unrelated comorbidities.

Bedridden patients, clinical trial participants, and those with a dual diagnosis of asthma and COPD (asthma–COPD overlap syndrome [ACOS]) were also excluded. Importantly, patient selection was performed independently of the research team to safeguard methodological integrity and avoid bias.

#### 2.2.3. Variables Collected

Data extracted from EHRs encompassed four main categories:

- Demographic variables: age and sex.
- Anthropometric variables: height, weight, and body mass index (BMI).
- Clinical variables: frequency of asthma exacerbations in the past 12 months (defined as any episode coded in the EHR as ‘acute bronchitis’ or ‘asthmatic exacerbation’), respiratory-related healthcare utilization (e.g., visits to primary care or emergency services), and treatment adherence indicators (see Section 3.1).
- Pharmacological variables: annual count of dispensed canisters of SAMAs and SABAs; rescue medication use patterns; maintenance therapy with inhaled corticosteroids combined with formoterol (inhaled corticosteroids [ICS]-formoterol); and systemic courses of corticosteroids and antibiotics.

A composite control variable was defined based on guideline-defined criteria (GINA 2025, GEMA 5.4, NICE 2024) and incorporates two key components: the occurrence of ≥1 documented asthma exacerbation and the use of ≥3 SABA canisters per year [7,8,17]. Patients meeting either criterion were classified as poorly controlled. This binary outcome provides a more pragmatic and guideline-aligned measure for identifying individuals who may be missed by narrower definitions of asthma control [23].

### 2.3. Clinical Guidelines and the Evidentiary Asymmetry Between SABAs and SAMAs

Despite transformative advances in asthma management, the oversight of SAMAs remains striking. Current guidelines—including GINA 2025, GEMA 5.4, and NICE 2024 —have decisively redefined the role of SABAs: monotherapy is no longer acceptable, overuse (≥3 canisters/year) is considered a clinical warning, and anti-inflammatory relievers (ICS-formoterol) are prioritized across all severity levels [7,8,17].

This paradigm shift stems from robust evidence—particularly from the SABINA program—demonstrating that SABA overuse increases exacerbation risk, worsens control, and raises mortality [12,13,14,15]. In contrast, SAMA use remains largely unexamined, unquantified, and unaddressed, both in clinical trials and in guidelines. No thresholds, no monitoring algorithms, and no specific recommendations exist beyond acute exacerbation management or rare β_2_-agonist intolerance. This asymmetry is not justified by data but by the absence of it.

Although SAMAs are routinely prescribed in real-world settings—often in outpatient care and sometimes as chronic reliever therapy—their long-term safety profile, impact on exacerbation burden, and predictive value remain unknown. This evidentiary void contrasts sharply with their widespread availability and prescriber familiarity. The result is a therapeutic blind spot: a drug class exempt from scrutiny despite fulfilling the same pharmacological role as SABAs—bronchodilation without inflammation control [9].

This study aims to address that gap. By quantifying the association between frequent SAMA use and key clinical outcomes in asthma—including exacerbations, healthcare utilization, and corticosteroid exposure—we seek to provide actionable evidence that could inform future guideline updates and challenge current assumptions about SAMA neutrality.

### 2.4. Statistical Analysis

All statistical analyses were conducted using R software (v4.3.4.2; The R Foundation; Austria). The primary outcome was the number of asthma exacerbations as a function of SAMA use. Variables with more than 50% of missing data were excluded from all analyses. The normality of continuous variables was assessed using the Shapiro–Wilk and D’Agostino–Pearson tests. Bivariate associations and potential collinearity were explored using Pearson’s correlation coefficient for normally distributed variables and Spearman’s rank correlation coefficient for non-normally distributed or ordinal variables.

Section 3.1 focuses on the binary classification of exacerbation risk (≥1 exacerbation) and follows a distinct methodological approach based on categorical comparisons. Chi-squared (*χ*^2^) tests were used to compare proportions between groups (SAMA users vs. non-users), and binary and ordinal logistic regression models were applied with a logit link function. Model calibration was assessed using the Hosmer–Lemeshow test, and overall fit was quantified with Nagelkerke’s pseudo-R^2^. Internal validation was performed using non-parametric bootstrap resampling with 1000 iterations. Additional concordance measures, including Somers’ D and Kendall’s τb, were reported where applicable. Pooled odds ratios were calculated using the Mantel–Haenszel method to account for stratified effects.

Section 3.2, Section 3.3, Section 3.4, Section 3.5, Section 3.6, Section 3.7, Section 3.8, Section 3.9 and Section 3.10 address count-based outcomes—including exacerbation frequency, respiratory-related healthcare utilization, systemic corticosteroid use, and antibiotic prescriptions—using generalized linear models (GLMs) with a log link function. Poisson, negative binomial (NB), zero-inflated Poisson (ZIP), and zero-inflated negative binomial (ZINB) models were evaluated. Model selection was based on theoretical fit, an assessment of overdispersion and zero inflation, and a comparison of Akaike Information Criterion (AIC) values. When parametric models failed to provide adequate fit or interpretability, a non-parametric model based on extreme gradient boosting (XGBoost) was assessed, though it was ultimately discarded due to inferior predictive performance.

The NB model was selected for most final analyses due to its favorable balance of fit (AIC), parsimony, interpretability, and robustness. Internal validation of coefficient stability was conducted via bootstrap resampling with 5000 iterations. Model diagnostics included estimates of dispersion (*θ*), multiple pseudo-R^2^ values (McFadden, Nagelkerke, Cox and Snell), and residual analysis. In Section 3.4, interaction effects are specifically assessed using a multiplicative term (SAMA × SABA) within the NB model. Multicollinearity is evaluated using variance inflation factors (VIFs), all of which are below 1.20. Where applicable, Mantel–Haenszel pooled odds ratios are reported to account for stratified effects.

A simulation-based power analysis was performed for each final model to estimate the probability of detecting the observed effect sizes with the available sample size (*n* = 132). For the primary model examining SAMA use and exacerbation frequency, the estimated power exceeded 80%. Power estimates for secondary outcomes are detailed in the results section. All statistical tests are two-tailed, with significance defined as *p* < 0.05 unless otherwise stated.

## 3. Results

### 3.1. Frequent SAMA Use Is Strongly Associated with Asthma Exacerbations

**Figure 1:**
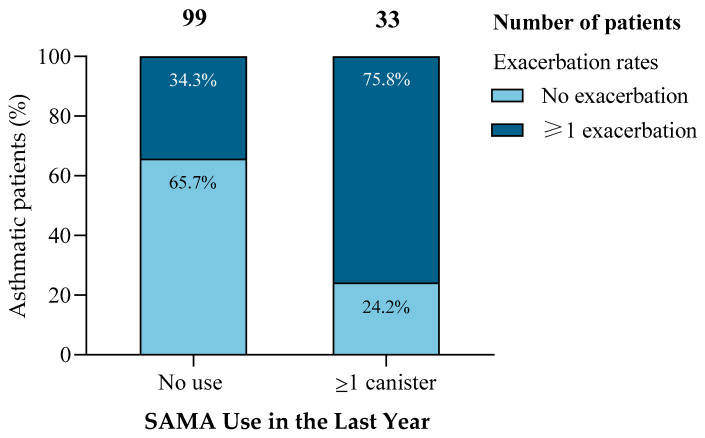
Increased exacerbation risk among patients with SAMA use. Patients prescribed ≥1 SAMA canister in the past year had significantly higher exacerbation rates (75.8%) than non-users (34.3%), while the proportion of patients without exacerbations was markedly lower (24.2% vs. 65.7%). This association was statistically significant (*χ*^2^ = 17.173, df = 1, *p* < 0.001).

Among the 132 patients analyzed, those prescribed at least one SAMA canister in the previous year (*n* = 33) experienced significantly more exacerbations than non-users (75.8% vs. 34.3%; *χ*^2^ = 17.173, *p* < 0.001) (Figure 1). This pattern was consistent across multiple statistical methods.

Binary and ordinal logistic regression yielded a consistent and significant association, with an odds ratio (OR) of 5.97 (95% CI: 2.43–14.66), indicating that SAMA users were nearly six times more likely to experience ≥1 exacerbation. Bootstrap validation (1000 iterations) produced a slightly more conservative but overlapping confidence interval (2.19–14.66), confirming the robustness of the estimate.

Model performance metrics improved with the inclusion of SAMA use: Nagelkerke’s R^2^ = 0.185, Hosmer–Lemeshow *p* = 0.500. Directional statistics further supported the association (Somers’ *d* = 0.357, Kendall’s τb = 0.361; both *p* < 0.001). The Mantel–Haenszel pooled OR replicated the primary finding (OR = 5.97; 95% CI: 2.43–14.66).

These results highlight a striking real-world discrepancy: although current guidelines restrict SAMA to acute emergency use [7,8,17], its chronic prescription appears common and is associated with clinically significant risk.

While the observational nature of the study precludes causal inference, the strength and consistency of the association suggest that frequent SAMA use may act as a proxy marker of uncontrolled asthma, potentially reflecting therapeutic inertia, insufficient anti-inflammatory coverage, or alternative prescribing preferences in select clinical profiles.

This finding warrants prospective validation and supports incorporating SAMA tracking into asthma control frameworks.

The following section explores whether this association displays a dose–response pattern and whether a clinically meaningful threshold for SAMA overuse can be identified.

### 3.2. Exacerbation Frequency Increases with SAMA Use in a Dose–Response Pattern

To assess whether higher SAMA exposure predicts an increased exacerbation burden, we applied a series of count-based regression models to examine the dose–response relationship.

Both variables displayed marked non-normality and overdispersion (Shapiro–Wilk and D’Agostino–Pearson *p* < 0.001), justifying the use of generalized count models. Correlation analyses revealed a moderate, statistically significant association between annual SAMA use and exacerbation frequency (Spearman’s ρ = 0.362; Pearson’s *r* = 0.370; both *p* < 0.001), along with substantial zero inflation.

Six candidate models were evaluated—linear, quantile, Poisson, NB, ZIP, and ZINB. The NB model demonstrated the best trade-off between goodness of fit (AIC = 317.83), coefficient stability, and clinical interpretability. While ZIP and ZINB yielded slightly lower AICs, they presented unstable estimates with wide confidence intervals.

**Figure 2:**
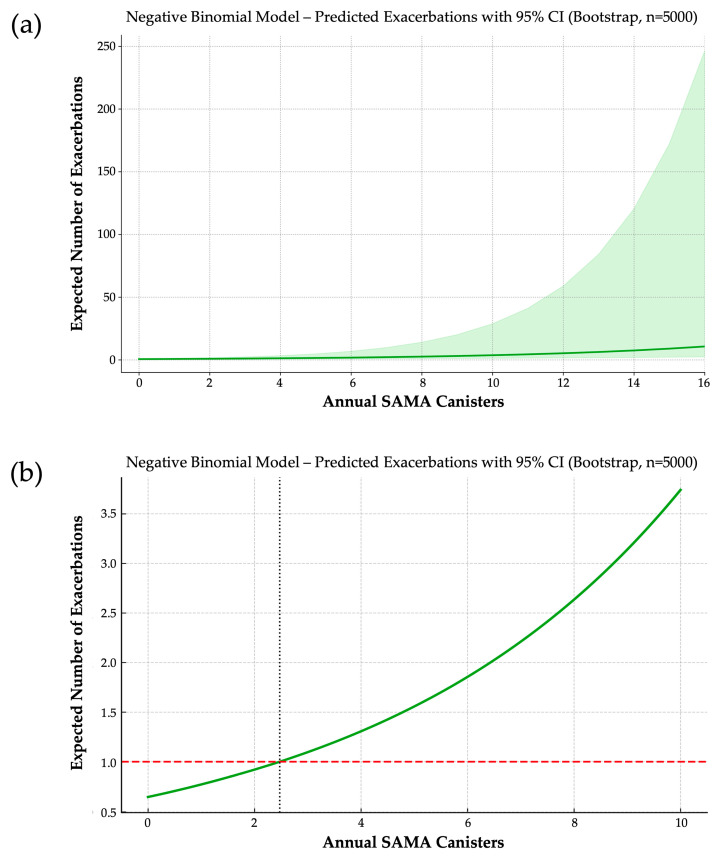
(**a**) Dose–response relationship between annual SAMA use and predicted exacerbation frequency. Based on a negative binomial model with 5000 bootstrap iterations, each additional SAMA canister per year is associated with a 19.2% increase in expected exacerbations. The solid line represents predicted values; the shaded area indicates the 95% confidence interval. (**b**) Predicted annual exacerbation rate by SAMA exposure and identification of a clinical threshold. A negative binomial model estimates exacerbation risk across increasing annual SAMA use. The horizontal dashed line marks one exacerbation per year—a widely accepted threshold of poor asthma control. The vertical dotted line indicates the inflection point (~2.5 canisters/year) beyond which predicted exacerbations exceed one, suggesting a clinically actionable cutoff.

In the final NB model, each additional SAMA canister per year was associated with a 19.2% increase in expected exacerbation frequency (IRR = 1.192; 95% CI: 1.107–1.430; bootstrap coefficient = 0.1753) (Figure 2a). For clinical reference, patients using five and ten canisters annually were predicted to experience approximately 2.4 and 5.8 times more exacerbations, respectively, than non-users.

The final model equation was the following:

ln(E[Exacerbations])=−0.4335+(0.1753·SAMA) ,

$$E\left[Exacerbations\right]=e^{-0.4335+(0.1753\cdot SAMA)}=0.6482\cdot {\left(1.1916\right)}^{SAMA}.$$

This formulation allows a direct estimation of exacerbation risk across the full spectrum of SAMA exposure.

From this model, we derived a clinically actionable threshold: the expected number of exacerbations surpasses one per year—a commonly accepted marker of poor asthma control—at approximately 2.5 SAMA canisters/year (Figure 2b). This inflection point suggests a shift from occasional reliever use to a pattern reflecting underlying disease instability.

This threshold may serve as a pragmatic marker in clinical practice to identify patients who warrant the following:

- Closer monitoring;
- Escalation or reassessment of maintenance therapy;
- Evaluation of adherence or phenotypic mismatch.

Furthermore, it provides a foundation for developing real-time EHR-based alerts, enabling earlier interventions and more targeted asthma care. Notably, while similar thresholds exist for SABAs, no such reference has been established for SAMAs. This study presents the first empirically validated cutoff, which could inform updates to clinical guidelines and risk stratification tools.

Model calibration was acceptable, with McFadden’s pseudo-R^2^ = 0.0365 and dispersion parameter *θ* = 1.7211, supporting the model’s appropriateness. Bootstrap validation (5000 iterations) confirmed the stability and reliability of estimates.

To confirm the adequacy of the sample size, a simulation-based power analysis was conducted. Estimated power to detect the observed effect was 75.6% at *n* = 100, 83.8% at *n* = 132 (actual sample), and 94.0% at *n* = 200.

These results support that the study was adequately powered to detect a clinically meaningful association between SAMA use and exacerbation frequency.

### 3.3. SABA Use and Exacerbation Frequency: A Modest but Clinically Relevant Association

To evaluate whether SABA use predicts asthma exacerbation frequency, we applied the same count-based modeling strategy used for SAMA analysis. The distribution of both variables exhibited marked non-normality and overdispersion (Shapiro–Wilk and D’Agostino–Pearson *p* < 0.001), justifying the use of NB regression.

Preliminary correlation analyses showed a weak but consistent trend (Spearman’s ρ = 0.141, *p* = 0.106; Pearson’s *r* = 0.162, *p* = 0.063), suggesting a potential association between higher SABA exposure and exacerbation burden. Among the six models tested—linear, quantile, Poisson, NB, ZIP, and ZINB—the NB model was selected for its optimal balance of statistical fit, coefficient stability, and interpretability. Poisson-based models were excluded due to overdispersion (Pearson *χ*^2^/df = 1.29), while ZIP and ZINB showed unstable inflation components and wide bootstrap intervals.

In the final NB model, SABA use emerged as a statistically significant predictor, with a regression coefficient (*β*) of 0.0591 (*p* = 0.0487; 95% CI: 0.0038–0.1280). This translates into an IRR of 1.061 (95% CI: 1.004–1.137), indicating that each additional SABA canister per year was associated with a 6.1% increase in expected exacerbations.

The fitted model equation was as follows:

ln(E[Exacerbations])=−0.3952+(0.0591·SABA) ,

$$E\left[Exacerbations\right]=e^{-0.3952+(0.0591\cdot SABA)}=0.6735\cdot {\left(1.0609\right)}^{SABA}.$$

For illustrative purposes, patients using five and ten SABA canisters annually were predicted to experience approximately 0.91 and 1.22 exacerbations per year, respectively, compared to 0.67 for non-users.

**Figure 3:**
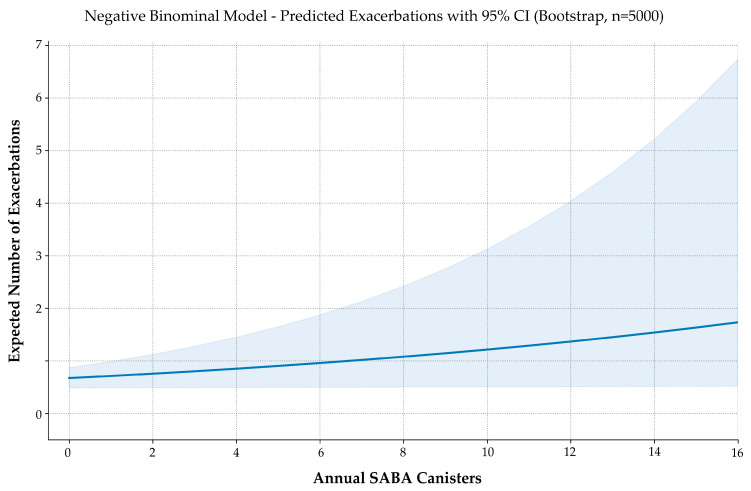
Predicted exacerbation frequency by annual SABA use. Negative binomial regression with 5000 bootstrap iterations estimates the expected number of exacerbations across increasing SABA canister use. The solid line represents model-predicted values; the shaded area indicates the 95% confidence interval. Although modest in magnitude, the model reveals a consistent upward trend in exacerbation risk with higher SABA exposure.

Model performance was acceptable, with a dispersion parameter (θ = 1.293) confirming moderate overdispersion. Bootstrap validation (5000 iterations) produced stable estimates and narrow confidence intervals (Figure 3). Diagnostic plots showed no violations of model assumptions. McFadden’s pseudo-R^2^ was 0.0101—low yet consistent with expected ranges for clinical count data.

Although modest in magnitude, the association is biologically plausible, statistically robust, and consistent with current guideline concerns about excessive SABA reliance. These findings support the concept of SABA use as a potential digital biomarker of suboptimal asthma control, reinforcing the role of controller-based therapy and prescribing surveillance.

To assess the sensitivity of the model, a simulation-based power analysis was conducted. With 5000 simulated datasets per sample size, the probability of detecting a significant SABA coefficient at *α* = 0.05 was estimated at 39.9% for *n* = 100, 46.8% for *n* = 132 (actual study size), 51.4% for *n* = 150, and 62.8% for *n* = 200.

These results indicate that while the observed association is likely real, larger cohorts are needed to confirm the effect with high statistical confidence and to support its routine use in predictive modeling frameworks.

### 3.4. Comparative Modeling of SAMA and SABA Use as Predictors of Exacerbation Risk

To formally characterize the differential predictive behavior of short-acting bronchodilators, we developed a negative binomial regression model incorporating annual canister counts of SABAs and SAMAs, along with a multiplicative interaction term. This framework allowed the simultaneous estimation of independent and joint risk gradients associated with reliever use.

The model was specified as follows:

$$\ln\left(E\left[Exacerbations\right]\right)=\beta_{0}+\left(\beta_{1}\cdot SABA\right)+\left(\beta_{2}\cdot SAMA\right)+\beta_{3}\cdot \left(SABA\times SAMA\right),$$

where SABA and SAMA represent the annual number of dispensed canisters for each inhaler type.

Detailed coefficient estimates and incidence rate ratios are presented in Table 1, while model diagnostics and comparative performance metrics are summarized in Table 2. Each additional SABA canister was associated with a 5.6% increase in the expected exacerbation rate (IRR = 1.056; 95% CI: 0.995–1.117; *p* = 0.0551), whereas SAMA canisters were linked to a significantly steeper 15.3% increase (IRR = 1.153; 95% CI: 1.052–1.267; *p* < 0.001). The interaction term suggested a modest but consistent additive risk contribution when both agents were used concurrently (IRR = 1.036; 95% CI: 0.999–1.075; *p* = 0.0527), with no differential baseline risk at zero canister use (intercept *p* < 0.0001).

**Figure 4:**
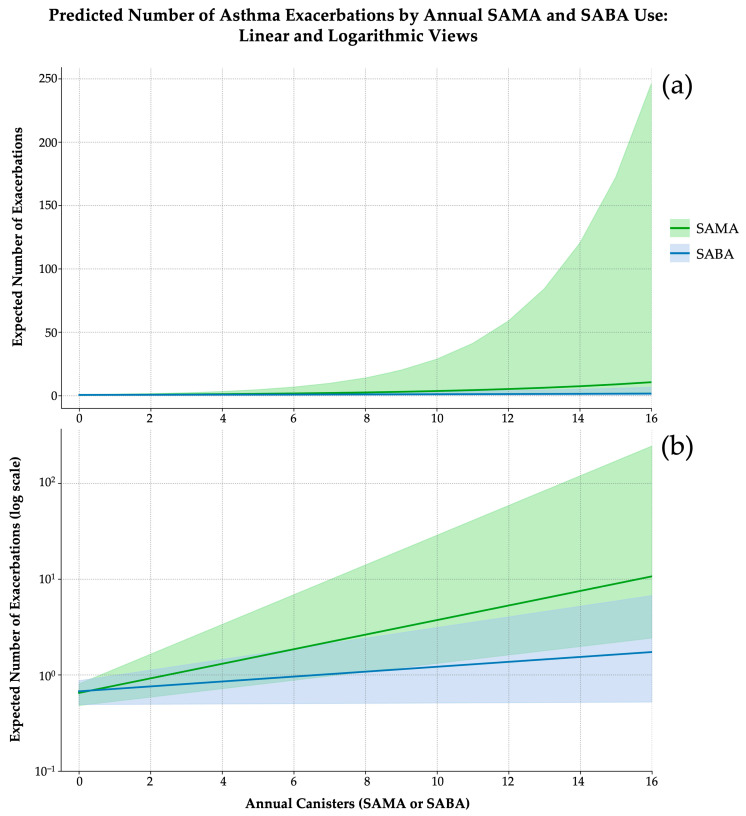
Predicted number of asthma exacerbations according to the annual canister use SAMAsSABAs, derived from separate negative binomial regression models (5000 bootstrap iterations). (**a**) Linear scale representation. SABAs (blue line) exhibit a mild, near-linear dose–response curve, with narrow and stable confidence intervals. In contrast, SAMAs (green line) demonstrate an exponential risk increase beyond 10 canisters/year, accompanied by substantial uncertainty at higher exposure levels. (**b**) Logarithmic Y-axis representation. This scale emphasizes the multiplicative nature of exacerbation risk. While both curves begin at comparable baseline values (~0.6–0.7 exacerbations/year), the SAMA curve diverges steeply, indicating a faster acceleration of risk. Shaded bands represent 95% confidence intervals.

Model fit improved with the inclusion of the interaction term, as evidenced by a greater log-likelihood (–151.53 vs. –160.18), lower AIC (313.05 vs. 326), and a dispersion estimate consistent with well-calibrated count data (*θ* = 2.974). Although the likelihood ratio test for the interaction term did not reach conventional statistical significance (*χ*^2^ = 3.32; df = 1; *p* = 0.0683), the observed trend and improved model performance supported its inclusion for explanatory purposes. Bootstrap validation (*n* = 5000) confirmed coefficient stability, and variance inflation factors remained below 1.20 for all predictors (Figure 4a).

These findings underscore the prognostic non-equivalence of SABAs and SAMAs. While SABA use followed a modest, near-linear trend, SAMA exposure appeared non-linear (Figure 4b). The interaction term further suggests additive risk amplification when both bronchodilators are used concurrently. Collectively, these results support the need for agent-specific thresholds in exacerbation risk stratification and suggest that SAMA exposure may function not only as a marker of instability but as a risk amplifier warranting heightened clinical vigilance.

### 3.5. SAMA and SABA Use Are Statistically Independent: Implications for Predictive Modeling and Clinical Practice

Understanding the relationship between SAMAs and SABAs is essential for refining asthma rescue strategies and improving the interpretability of inhaler-based digital biomarkers. Although both agents are bronchodilators and are frequently categorized as ‘rescue medications’, their real-world usage patterns may reflect distinct clinical rationales.

To assess whether SAMA use predicts SABA dispensing behavior, we applied a multimethod analytical approach, combining classical statistical techniques and machine learning algorithms.

Correlation analysis showed no significant association between annual SAMA and SABA use. Spearman’s ρ indicated a weak, non-significant inverse trend (*ρ* = −0.145, *p* = 0.097), and linear regression confirmed the absence of a predictive value (*β* = −0.183, *p* = 0.127; R^2^ = 0.009). Higher-order polynomial regressions, including cubic models, failed to improve the explanatory power.

Count-based analyses yielded consistent results. A negative binomial regression model produced a non-significant SAMA coefficient (*β* = −0.1434; 95% CI: −0.4754 to 0.0249; *p* = 0.124; AIC = 506.3), while a zero-inflated negative binomial model demonstrated no effect in either the count or inflation components (all *p* > 0.7).

To ensure robustness, we conducted 5000-sample bootstrap simulations, which confirmed the absence of stable associations. An additional extreme gradient boosting (XGBoost) model was tested to explore potential nonlinearities or interaction effects. However, it showed poorer predictive performance than the previously evaluated statistical models and failed to improve model fit. Given its limited discriminative capacity and lack of added value, the model was discarded.

### 3.6. Predictive Model of Exacerbation Risk Based on Rescue Medication Burden

**Figure 5:**
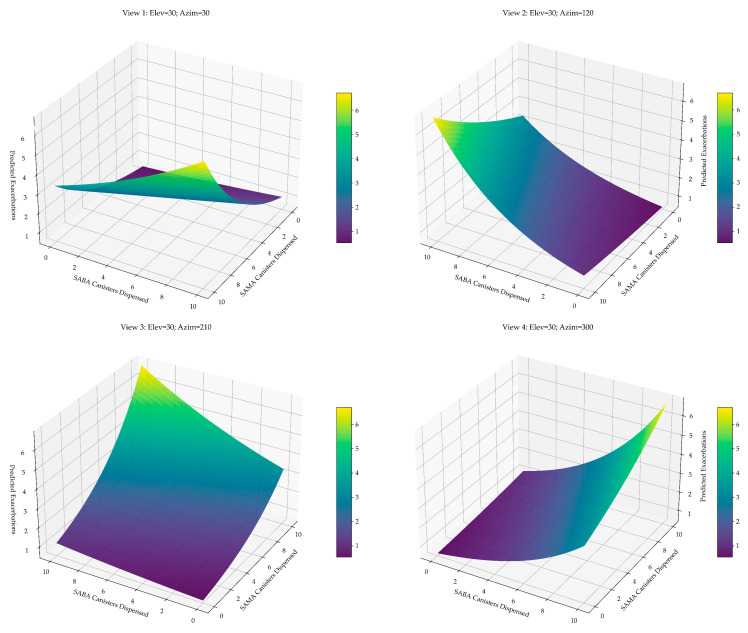
Predicted exacerbation surface based on annual SAMA and SABA use. Three-dimensional surface plots derived from the negative binomial model illustrate the expected number of exacerbations as a function of combined SAMA and SABA exposure. The plots highlight the asymmetric and multiplicative nature of the relationship: exacerbation risk increases with both agents but rises more steeply with SAMAs, underscoring its role as a sensitive marker of asthma instability.

To evaluate the joint predictive value of short-acting bronchodilator use on asthma exacerbations, we developed a multivariable model incorporating annual SAMA and SABA canister dispensing (Figure 5).

Bivariate analyses confirmed a moderate, statistically significant association for SAMA use (Pearson’s *r* = 0.370; Spearman’s ρ = 0.362; both *p* < 0.001), with stable bootstrap estimates. In contrast, SABA use showed a weaker, non-significant trend (Pearson’s *r* = 0.162, *p* = 0.063; Spearman’s ρ = 0.141, *p* = 0.106), with confidence intervals crossing the null.

Due to overdispersion in the count outcome (variance-to-mean ratio > 2), we tested several candidate models. A negative binomial regression was selected for its balance of fit, coefficient stability, and interpretability. Although zero-inflated models yielded marginally lower AIC values, they were excluded due to unstable estimates and wide bootstrap intervals.

The final model, validated through 5000 bootstrap iterations, included both predictors and was expressed as follows:

$$E[Exacerbations]=e^{\left[-0.6337+\left(0.1839\;\cdot \;SAMA\right)+\left(0.0709\;\cdot \;SABA\right)\right]}\;,$$

$$E[Exacerbations]=0.5306\cdot \;1.2019^{SAMA}\cdot 1.0735^{SABA}.$$

Each additional SAMA canister was associated with a 20.2% increase in expected exacerbations (IRR = 1.202; 95% CI: 1.122–1.421) and each SABA canister with a 7.3% increase (IRR = 1.073; 95% CI: 1.019–1.144). The effect of SAMAs was approximately 2.77 times greater, indicating that one SAMA canister confers a similar exacerbation risk to nearly three SABA canisters.

The model estimated a baseline exacerbation rate of 0.53 per year in patients not using rescue medication. For a patient using three SAMA and five SABA canisters annually, the expected rate increased to 1.31/year—a 2.5-fold elevation in risk.

To confirm the reliability of these estimates, a simulation-based power analysis was conducted using 5000 iterations per sample size. The model demonstrated a power of 80.0% at *n* = 100, 88.2% at *n* = 132 (study sample), and 96.8% at *n* = 200, confirming that the sample size was sufficient to detect clinically meaningful associations with both predictors.

### 3.7. Increased SAMA Use Predicts Higher Respiratory Healthcare Utilization

To determine whether SAMA use correlates with increased healthcare demand, we modeled the relationship between annual SAMA dispensing and the number of respiratory-related primary care consultations.

The outcome variable exhibited strong right-skew and overdispersion (mean = 2.67; SD = 2.97), justifying the use of NB regression over Poisson. Zero-inflated models were also considered, given the high proportion of zero-consultation observations.

Although the ZINB model showed slightly improved fit (AIC = 555.27; log-likelihood = −272.63; pseudo-R^2^ = 0.0339), the inflation component related to SAMA use was not statistically significant (*p* = 0.776), reducing its interpretive value. By contrast, the NB model provided better parsimony, coefficient stability, and clinical clarity and was therefore selected as the final model.

The fitted regression equation was the following:

$$E[Consultations]=e^{\left[0.8217+\left(0.1553\;\cdot \;SAMA\right)\right]}\;,$$

$$E\left[Consultations\right]=2.2744\cdot 1.1680^{SAMA}\;.$$

Each additional SAMA canister was associated with a 16.8% increase in expected respiratory consultations (IRR = 1.168; 95% CI: 1.090–1.331; *p* < 0.001).

**Figure 6:**
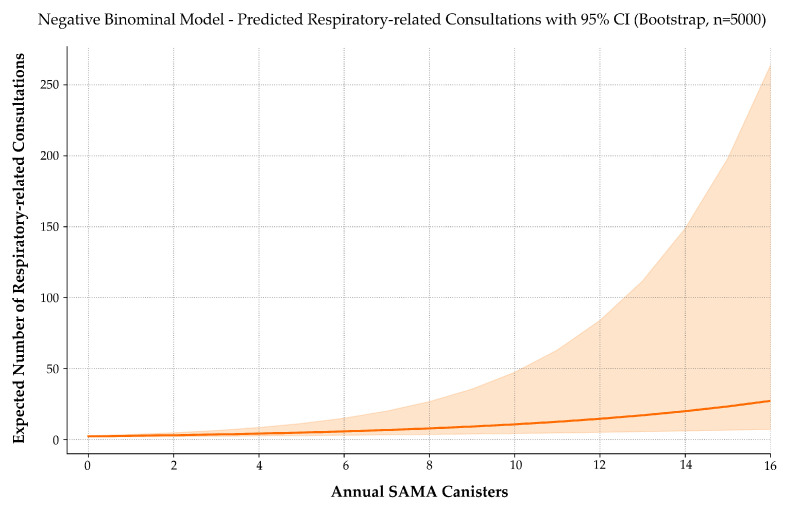
Predicted number of respiratory-related consultations by annual SAMA use. A negative binomial model with 5000 bootstrap iterations estimates the expected number of asthma-related consultations as a function of annual SAMA canister use. The solid line represents predicted values; the shaded area indicates the 95% confidence interval. Consultation frequency rises progressively with SAMA use, suggesting a link between reliever reliance and healthcare demand. The widening confidence band at higher doses reflects increased variability due to fewer observations in that range.

To assess the model’s reliability, a simulation-based power analysis was conducted using 5000 replications per sample size (Figure 6). The estimated power to detect the observed effect was 74.3% at *n* = 100, 85.1% at *n* = 132 (actual sample size), and 94.6% at *n* = 200.

These values confirm sufficient statistical sensitivity at the current sample size and reinforce the robustness of the effect.

The NB model thus offers a clinically interpretable, statistically robust framework that supports the inclusion of SAMA use as a proxy for real-world disease impact. This reinforces its utility not only in predicting exacerbation risk but also in forecasting resource use, making it a candidate variable for incorporation into population-level asthma surveillance systems and EHR-based alert algorithms.

### 3.8. SAMA Use Is Associated with Increased Systemic Corticosteroid Prescriptions

**Figure 7:**
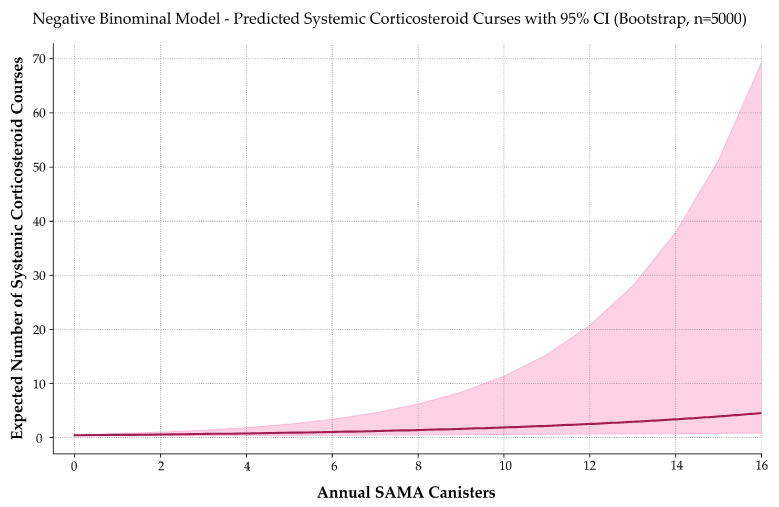
Predicted systemic corticosteroid use by annual SAMA dispensing. A negative binomial model estimates the expected number of oral corticosteroid courses as a function of annual SAMA canister use. The solid curve represents predicted values; the shaded band indicates the 95% confidence interval. Corticosteroid use increases exponentially with SAMA exposure, reinforcing its role as a marker of poor asthma control and potential treatment escalation need.

To assess whether frequent SAMA use correlates with systemic corticosteroid (OCS) exposure, we modeled the number of annual OCS prescriptions using negative binomial regression (Figure 7). This model was selected based on its superior handling of overdispersion, consistent coefficient behavior, and clinical interpretability.

The fitted model was expressed as follows:

$$E[Systemic\;corticosteroids\;courses]=e^{\left[-0.8419+\left(0.1470\;\cdot \;SAMA\right)\right]}\;,$$

$$E\left[Systemic\;corticosteroids\;courses\right]=0.4309\cdot 1.1584^{SAMA}.$$

Each additional SAMA canister was associated with a 15.8% increase in the expected number of OCS prescriptions per year (IRR = 1.158; 95% CI: 1.063–1.352; *p* < 0.001). Normality testing confirmed non-Gaussian distributions (*p* < 0.001), and Spearman’s correlation indicated a moderate, statistically significant relationship (*ρ* = 0.335; *p* < 0.001).

Although a zero-inflated Poisson model produced a slightly lower AIC, it offered no clear advantage in interpretability or coefficient stability and was therefore not selected.

To evaluate the reliability of this association, a simulation-based power analysis was conducted using 5000 iterations per sample size. Power to detect the observed effect was estimated at 64.5% for *n* = 100, 74.1% for *n* = 132 (study sample), and 86.9% for *n* = 200.

Although the current sample yielded power slightly below the conventional 80% threshold, the upward trend and narrow confidence intervals support the robustness of the finding and suggest that confirmatory evidence may emerge from larger prospective cohorts.

### 3.9. SAMA Use Is Associated with Increased Respiratory Antibiotic Prescriptions

To evaluate whether SAMA use predicts the frequency of respiratory-related antibiotic prescriptions, we applied a series of count-based regression models.

Both variables exhibited significant non-normality (Shapiro–Wilk *p* < 0.001), warranting non-parametric assessment. Spearman’s correlation showed a weak-to-moderate, statistically significant association between SAMA use and antibiotic prescriptions (*ρ* = 0.277; *p* = 0.0013).

**Figure 8:**
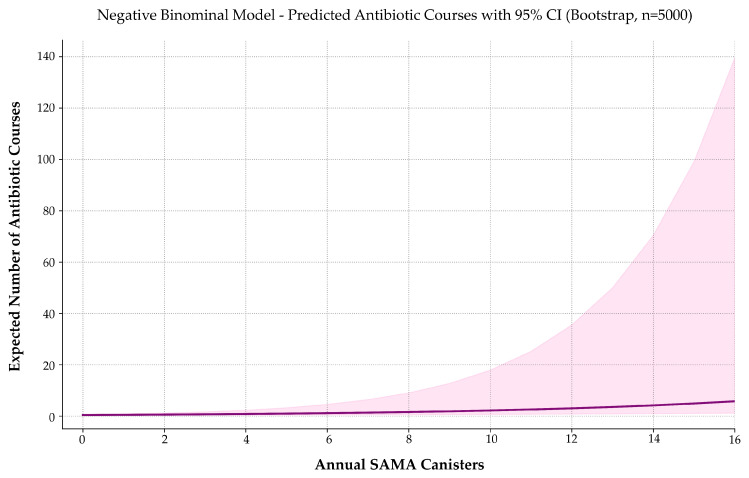
Predicted number of respiratory-related antibiotic prescriptions by annual SAMA use. A negative binomial model estimates expected annual antibiotic use as a function of SAMA canister dispensing. The solid curve represents predicted values; the shaded band indicates the 95% confidence interval. Antibiotic prescriptions increase progressively with SAMA exposure, suggesting a potential association with higher respiratory morbidity or symptom burden in asthma management.

Among the tested models, negative binomial regression offered the best trade-off between fit quality, overdispersion control, and bootstrap stability (Figure 8). Zero-inflated models marginally improved AIC, but their loss of coefficient significance compromised interpretability and clinical utility.

The final model was expressed as follows:

$$E\left[Antibiotic\;courses\right]=e^{\left[-0.8288+\left(0.1613\;\cdot \;SAMA\right)\right]}\;,$$

$$E\left[Antibiotic\;courses\right]=0.4366\cdot {1.1750\;}^{SAMA}\;.$$

Each additional SAMA canister was associated with a 17.5% increase in expected antibiotic prescriptions (IRR = 1.175; 95% CI: 1.089–1.408; *p* = 0.0053). The model intercept was also statistically significant (*p* < 0.001), indicating a baseline antibiotic rate of approximately 0.44 courses per year in non-users.

These results suggest that increased SAMA use may reflect higher respiratory morbidity or symptom burden, reinforcing its potential role as a proxy for healthcare utilization beyond exacerbations.

To assess the model’s sensitivity, a simulation-based power analysis was conducted using 5000 iterations per sample size. The estimated power to detect the observed effect was 54.6% at *n* = 100, 63.8% at *n* = 132 (actual sample size), and 79.9% at *n* = 200.

Although the current sample approaches the conventional 80% threshold, the upward trend and narrow confidence intervals support the stability of the finding. Larger cohorts would increase inferential certainty and support broader generalizability.

From a clinical perspective, the association between SAMA use and antibiotic exposure supports its interpretation as a surrogate marker of disease instability, with potential application in risk stratification and early intervention strategies.

### 3.10. SAMA Use Is Not Significantly Associated with Maintenance Therapy Gaps

To examine whether the increased use of SAMA reflects poor adherence to inhaled maintenance therapy, we assessed its relationship with three operational indicators of adherence: (1) the number of days without treatment due to an absent prescription, (2) the number of days without therapy despite an active prescription, and (3) the total number of days combining both gaps during the 12-month observation window. As none of these variables followed a normal distribution (Shapiro–Wilk *p* < 0.001), Spearman’s rank correlation was used to evaluate the strength and direction of associations. Across all three indicators, correlations with SAMA use were weak and statistically non-significant: ρ = 0.097 (*p* = 0.269) for prescription gaps; *ρ* = −0.031 (*p* = 0.727) for non-collection; and *ρ* = 0.125 (*p* = 0.153) for the combined measure.

No significant association was observed between SAMA use and interruptions in maintenance therapy, regardless of whether those interruptions resulted from prescriber-related or patient-related factors.

## 4. Discussion

### 4.1. Key Findings and Conceptual Implications

This study offers the first comprehensive assessment of SAMA use in asthma under routine clinical conditions. The findings challenge the historical assumption of therapeutic neutrality attributed to SAMAs. Rather than functioning as a benign alternative to SABAs, frequent SAMA use emerged as a robust and independent marker of clinical instability, associated with increased exacerbation risk, higher healthcare utilization, and greater systemic corticosteroid exposure. These associations were statistically consistent and clinically actionable, underscoring the need to minimize avoidable prescriptions of both SAMAs and SABAs.

While SABA overuse has been extensively addressed in clinical guidelines, the role of SAMAs has remained largely unexamined. This study highlights an under-recognized dimension of reliever overuse within a primary care population. Although based on a single-country cohort of 132 patients, the consistency of the associations justifies prospective validation in broader and more heterogeneous populations.

This analysis also addresses a conceptual gap in the current therapeutic framework. Recent guideline updates—GINA 2025, GEMA 5.4, and NICE 2024—discourage SABA monotherapy and promote anti-inflammatory reliever strategies [7,8,17]. In contrast, SAMAs remain absent from these frameworks [7,8,17]. Their continued omission may reflect structural oversight rather than clinical prudence, contributing to the under-recognition of risk and delayed intervention in patients who rely on SAMAs as their primary reliever.

### 4.2. Mechanistic Plausibility and Digital Biomarker Potential

The quantitative associations identified in this study are consistent and clinically meaningful. Patients prescribed SAMAs exhibited a nearly sixfold increase in the odds of experiencing exacerbations. Each additional canister was associated with increases of 16.8% in respiratory consultations, 15.8% in systemic corticosteroid use, and 17.5% in antibiotic prescriptions. These findings suggest that SAMA use is not merely a response to symptoms but a marker of persistent clinical instability.

The biological plausibility of these associations is well supported. Muscarinic antagonists provide short-term bronchodilation but fail to address airway inflammation—the central mechanism in asthma pathophysiology [22,25,26,27]. When used in isolation, SAMAs may inadvertently reinforce disease persistence, particularly without concurrent controller therapy [26]. Additionally, their effects on mucociliary clearance and airway reactivity may contribute to adverse outcomes [28,29,30,31].

SAMA use meets key criteria for a digitally traceable and clinically relevant biomarker of asthma instability since it is readily captured in structured EHRs and shows consistent associations with outcomes of interest. Its correlation with systemic corticosteroid use suggests value for anticipating treatment escalation, while its relationship with antibiotic prescriptions may reflect higher respiratory morbidity.

Importantly, SAMA and SABA use demonstrated statistical and behavioral independence across multiple analytical approaches, including correlation analysis, negative binomial modeling, and machine learning. This challenges the assumption that these agents represent a shared pattern of reliever overuse. Instead, their use appears driven by distinct prescribing logic, potentially shaped by clinical phenotype, prescriber behavior, or institutional norms.

This distinction has implications for predictive modeling and digital health strategies. Treating SAMAs and SABAs as interchangeable may obscure their individual predictive value. Modeling them as independent variables, as implemented in the Seleida architecture [23], avoids collinearity and enables drug-specific interpretation thresholds. The absence of an association between SAMA use and maintenance therapy gaps further suggests that frequent SAMA use does not merely reflect poor adherence but may indicate therapeutic misalignment or phenotype-specific reliever reliance.

These insights reinforce the need for targeted investigation into the clinical drivers of SAMA overuse. Divergent prescribing patterns—potentially influenced by β_2_-agonist intolerance, prescriber preferences, or formulary restrictions—highlight the heterogeneity of real-world practice. Future research should explore whether SAMA use identifies a distinct asthma endotype or reflects a deviation from guideline-based care. Longitudinal studies incorporating biomarker profiles, lung function trends, treatment trajectories, and adherence metrics will be essential to disentangle these mechanisms and support personalized strategies in digital frameworks.

These findings also support the construction of composite indices of reliever use that retain the distinct contributions of SAMAs and SABAs. When integrated into predictive models or surveillance systems, such indices may enhance risk stratification by capturing divergent bronchodilator use patterns without conflating their predictive signals [23].

### 4.3. Clinical Implications and Integration into Asthma Guidelines

A key clinical insight from this study is the identification of a data-driven inflection point in SAMA use. Beyond approximately 2.5 canisters per year, the predicted exacerbation rate exceeds one annually, a widely accepted marker of poor asthma control. This threshold provides, for the first time, an operational definition of SAMA overuse and a pragmatic benchmark for clinical monitoring. It also lays the foundation for integration into treatment step-up algorithms, digital dashboards, and real-time alert systems.

Current asthma guidelines—including GINA 2025, GEMA 5.4, and NICE 2024—already specify formal thresholds for SABA overuse (e.g., ≥3 canisters/year) to identify patients at risk of deterioration [7,8,17,32,33,34]. In contrast, SAMAs remain unquantified and unmonitored, reflecting their omission from contemporary treatment frameworks rather than a deliberate clinical rationale. This oversight may unintentionally normalize chronic SAMA use and delay therapeutic reassessment in patients who depend on them as their primary reliever.

Structured EHR data can support the integration of SAMA-based metrics into clinical surveillance tools, risk stratification models, and population health platforms. The strength and consistency of the observed associations reinforce their potential as an independent indicator of poor control. Incorporating SAMAs into real-world decision-support systems could enhance the early identification of suboptimal control and prompt timely intervention.

Ultimately, the principle that symptom relief is not equivalent to disease control must apply to all short-acting bronchodilators. Prescribing a reliever—whether SABAs or SAMAs—without appropriate anti-inflammatory therapy reflects a systemic misalignment with current evidence and undermines efforts to achieve sustained asthma control [5,12,13,14,15,35,36,37].

### 4.4. Research Agenda and Path to Translation

The present findings support the recognition of both SAMAs and SABAs as quantifiable, EHR-traceable markers of exacerbation risk, with potential applications in clinical alerts, decision-support tools, and individualized care escalation. While SABA overuse is actively monitored in current guidelines—such as GINA 2025, GEMA 5.4, and NICE 2024 [7,8,17]—SAMAs remain largely unaddressed despite their frequent use in real-world outpatient care. This omission likely reflects a lack of formal evidence rather than an absence of risk.

Although causality cannot be inferred from this retrospective cross-sectional analysis, the magnitude, consistency, and biological plausibility of the observed associations provide a strong rationale for prospective validation. Future studies should incorporate objective biomarkers—such as blood eosinophils and fractional exhaled nitric oxide (FeNO)—as well as longitudinal lung function data and patient-reported outcomes to determine whether SAMA use not only reflects poor control but also signals specific inflammatory or clinical phenotypes.

Within this framework, priority should be given to large-scale validation studies and interventional trials assessing the impact of reducing or withdrawing chronic SAMA use. Digital phenotyping using clustering and machine learning techniques could help define high-risk reliever-user profiles, while implementation research may identify the organizational or behavioral barriers that sustain inappropriate prescribing. Additionally, qualitative investigations into clinician decision-making, patient preferences, and institutional protocols will be essential to translate observational insights into effective practice change.

Understanding these multidimensional drivers will be key to integrating SAMA-based indicators into routine care and narrowing the gap between evidence and clinical decision-making.

### 4.5. Strengths and Limitations

This study has several strengths. It was conducted in a real-world primary care setting using EHRs from National Health System centers, enhancing external validity and clinical relevance. The analytical approach was rigorous and reproducible, incorporating advanced techniques such as bootstrap resampling, zero-inflated modeling, and simulation-based power analyses. The outcomes assessed were directly relevant to asthma control, and the proposed thresholds are pragmatically defined and potentially suitable for integration into digital health tools.

However, several limitations must be considered. First, the relatively small sample size—although adequate for a pilot analysis—limits statistical power and generalizability. The study population was also demographically homogeneous and drawn exclusively from a single country, which may constrain extrapolation to settings with different prescribing norms or clinical practices. While effect sizes remained robust under resampling, validation in larger, more diverse cohorts is needed to confirm external applicability. Second, the absence of spirometric data may have introduced diagnostic imprecision. Although clinical diagnostic criteria were rigorously applied, the lack of objective measures—such as bronchodilator reversibility or FeNO—may have led to misclassification, particularly in patients with atypical phenotypes. This limitation reflects a broader challenge in primary care, where access to pulmonary function testing is often limited [38,39,40]. Third, the retrospective design and reliance on EHR data introduce potential biases related to incomplete documentation, coding variability, and imprecise quantification of reliever use. Despite excluding variables with high rates of missingness, residual misclassification cannot be ruled out, underscoring the need for improved data standardization.

Additionally, the study did not differentiate between appropriate and inappropriate SABA use, nor did it capture key factors such as treatment adherence, self-management behavior, or the rationale behind prescribing decisions. Relevant confounders—including asthma phenotypes, disease severity, previous exacerbations, and inflammatory markers—were also not included and should be considered in future research. Finally, although the operational threshold for SAMA use offers a pragmatic benchmark for monitoring, it is derived from a single cohort and should be interpreted cautiously until validated externally. Despite these limitations, the study provides novel, real-world evidence linking frequent SAMA use to clinically meaningful indicators of asthma instability and supports the expansion of reliever monitoring beyond SABAs, particularly in digitally enabled or resource-constrained settings.

## 5. Conclusions

Although current asthma guidelines have progressed to discourage SABA monotherapy, they remain silent on SAMAs, leaving a critical dimension of reliever behavior unmonitored. This study demonstrates that SAMA use in asthma is not clinically inert. Rather, it emerges as a digitally traceable, clinically relevant, and under-recognized indicator of disease instability—currently absent from existing clinical guidelines.

Frequent SAMA dispensing was independently associated with increased exacerbation risk, greater healthcare utilization, and higher use of systemic corticosteroids and antibiotics—outcomes that were comparable to, and in the case of exacerbation risk, exceeded those traditionally attributed to SABA overuse.

Pending prospective validation, the identified threshold for SAMA use may serve as a foundation for future clinical tools, including integration into EHRs, clinical dashboards, and decision-support algorithms. SAMA prescribing should no longer be viewed as a neutral act but as a measurable signal of therapeutic drift—potentially modifiable through timely intervention.

While causality cannot be inferred from this retrospective design, the internal consistency of results across multiple outcomes, their biological plausibility, and the stability of estimates under extensive bootstrap resampling all support the need for prospective validation. Importantly, these findings should be regarded as preliminary. Their confirmation in larger, more diverse cohorts is essential to establish generalizability, reinforce external validity, and justify potential integration into clinical practice guidelines.

Incorporating SAMA tracking into asthma management offers an opportunity to anticipate instability before it becomes clinically manifest, shifting the paradigm from reaction to prevention and from symptomatic relief to sustained control.

## Figures and Tables

**Table 1:** Coefficients, CIs, and IRRs from the interaction model. Negative binomial model predicting the annual exacerbation frequency based on the number of canisters and drug type (SABAs vs. SAMAs), including the interaction term. IRRs are derived by exponentiating β coefficients.

Term	Estimate	Standard Error	Z Value	*p*-Value	CI Lower	CI Upper	IRR	IRR CI Lower	IRR CI Upper
(Intercept)	−0.6122	0.1514	−4.040	<0.0001 ***	−0.9208	−0.3198	0.542	0.398	0.726
SABA	0.0543	0.0283	1.918	0.0551	−0.0052	0.1105	1.056	0.995	1.117
SAMA	0.1424	0.0432	3.295	0.0010 **	0.0511	0.2370	1.153	1.052	1.267
SABA × SAMA	0.0354	0.0183	1.937	0.0527	−0.0006	0.0723	1.036	0.999	1.075

Statistically significant values are indicated by *** *p* < 0.0001 or ** *p* < 0.01.

**Table 2:** Performance comparison across negative binomial models. Comparison of SAMA-only, SABA-only, and joint models. Metrics include IRR, dispersion (*θ*), log-likelihood, AIC/BIC, and the qualitative interpretation of risk behavior.

Parameter	SAMA Model	SABA Model	Joint Model with Interaction
Estimate (β)	0.1753	0.0591	SABA: 0.0543 SAMA: 0.1424 Interaction (SABA × SAMA): 0.0354
IRR per canister	1.1916	1.0609	SABA: 1.056 SAMA: 1.153 Interaction (SABA × SAMA): 1.036
95% IC for IRR	1.107–1.430	1.004–1.137	SABA: [0.995, 1.117] SAMA: [1.052, 1.267] Interaction (SABA × SAMA): [0.999, 1.075]
*p*-value (predictor)	<0.0001	0.0487	SABA: 0.0551 SAMA: 0.0010 Interaction (SABA × SAMA): 0.0527
Dispersion (θ)	1.721	1.293	0.336
Log-likelihood	−155.916	−160.183	−151.526
AIC/BIC	317.83/326.48	326.37/335.01	313.05/327.47
Pseudo-R^2^ (Nagelkerke/McFadden)	0.0936/0.0365	0.0267/0.0101	0.1580/0.0636
Bootstrap validation	(n = 5000)	(n = 5000)	(n = 5000)
Clinical interpretation	Exponential increase in risk; high interindividual variability.	Linear–moderate increase; stable estimates.	Additive amplification of risk when both agents are used; dominant contribution from SAMAs.

## Data Availability

The original contributions presented in the study are included in the article; further inquiries can be directed to the corresponding authors.
